# Supplementary material for: Investigation of reversible histone acetylation and dynamics in gene expression regulation using 3D liver spheroid model
Source: Epigenetics Chromatin. 2022 Nov 21;15:35. doi: 10.1186/s13072-022-00470-7 (PMC9677638; doi:10.1186/s13072-022-00470-7)
Supplement: Supplementary file 1 — Additional file 1: Fig. S1. Spheroids express proteins characteristic of the human liver. (A) Size distribution of liver spheroids. For the proteome analysis, flat cells and spheroids were collected, processed and the peptides analyzed by mass spectrometry. Bar graphs show the relative abundance of (B) metallothioneins and (C) liver biomarkers. Data are represented as means ± SEM. Fig. S2. NaBut treatment induce histone hyperacetylation in flat cells. HepG2/C3A cells were treated with 20 mM NaBut and were kept in culture for 3 days. After treatment, histones were extracted and analyzed by mass spectrometry. (A) Total levels of histone peptides containing 1, 2 or 3 methylations (me1, me2, me3, respectively) or containing acetylations (ac). (B) Total levels of histone H4 peptides containing acetylations (1ac, 2ac, 3ac, 4ac). Unmod, unmodified peptide. (C) Relative abundance of histone H3 acetylated peptides. Data are represented as means ± SD. Nt, non-treated. (D) Volcano plot representing NaBut vs Nt fold change after 3 days of treatment. Fig. S3. Liver spheroids have a slow replication rate and can recover from treatment. (A) Adenylate kinase was measured following NaBut treatment. The culture supernatant from flat cells and spheroids were collected and analyzed by luminescence. (B) Relative abundance of DNMT1 in flat cells and spheroids. (C) Labeling incorporation in flat cells and spheroids. Data are represented as means ± SEM. * p < 0.05, ** p < 0.005, and *** p < 0.0005 when compared with flat cells. [file 13072_2022_470_MOESM1_ESM.docx]

**Investigation of reversible histone acetylation and dynamics in gene expression regulation using 3D liver spheroid model**

Stephanie Stransky, Ronald Cutler, Jennifer Aguilan, Edward Nieves, Simone Sidoli

**Fig. S1. Spheroids express proteins characteristic of the human liver. (A)** Size distribution of liver spheroids. For the proteome analysis, flat cells and spheroids were collected, processed and the peptides analyzed by mass spectrometry. Bar graphs show the relative abundance of **(B)** metallothioneins and **(C)** liver biomarkers. Data are represented as means ± SEM.

**Fig. S2. NaBut treatment induce histone hyperacetylation in flat cells.** HepG2/C3A cells were treated with 20 mM NaBut and were kept in culture for 3 days. After treatment, histones were extracted and analyzed by mass spectrometry. **(A)** Total levels of histone peptides containing 1, 2 or 3 methylations (me1, me2, me3, respectively) or containing acetylations (ac). **(B)** Total levels of histone H4 peptides containing acetylations (1ac, 2ac, 3ac, 4ac). Unmod, unmodified peptide. **(C)** Relative abundance of histone H3 acetylated peptides. Data are represented as means ± SD. Nt, non-treated. **(D)** Volcano plot representing NaBut vs Nt fold change after 3 days of treatment.

**
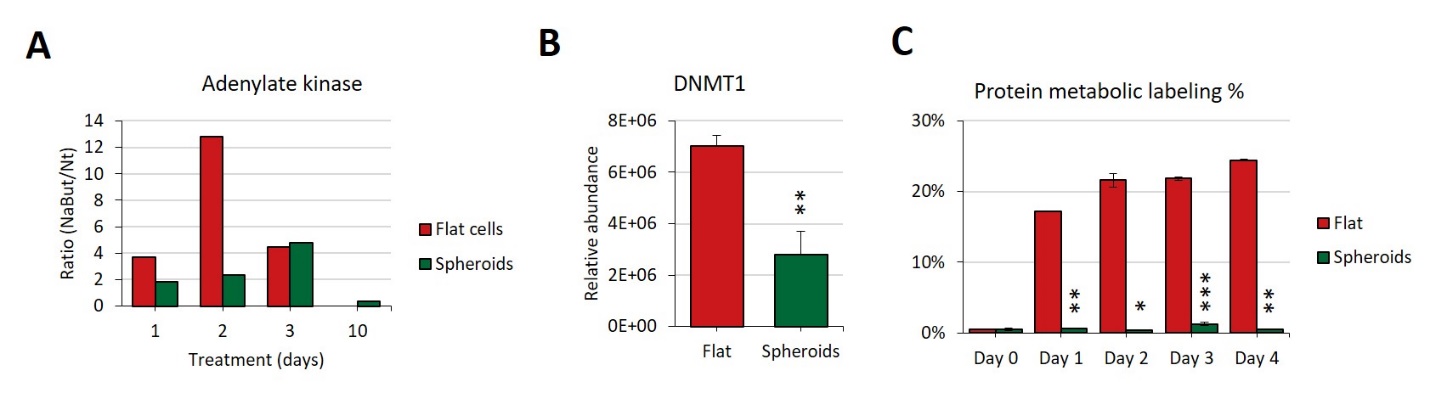
**

**Fig. S3. Liver spheroids have a slow replication rate and can recover from treatment. (A)** Adenylate kinase was measured following NaBut treatment. The culture supernatant from flat cells and spheroids were collected and analyzed by luminescence. **(B)** Relative abundance of DNMT1 in flat cells and spheroids. **(C)** Labeling incorporation in flat cells and spheroids. Data are represented as means ± SEM. * *p* < 0.05, ** *p* < 0.005, and *** *p* < 0.0005 when compared with flat cells.

**
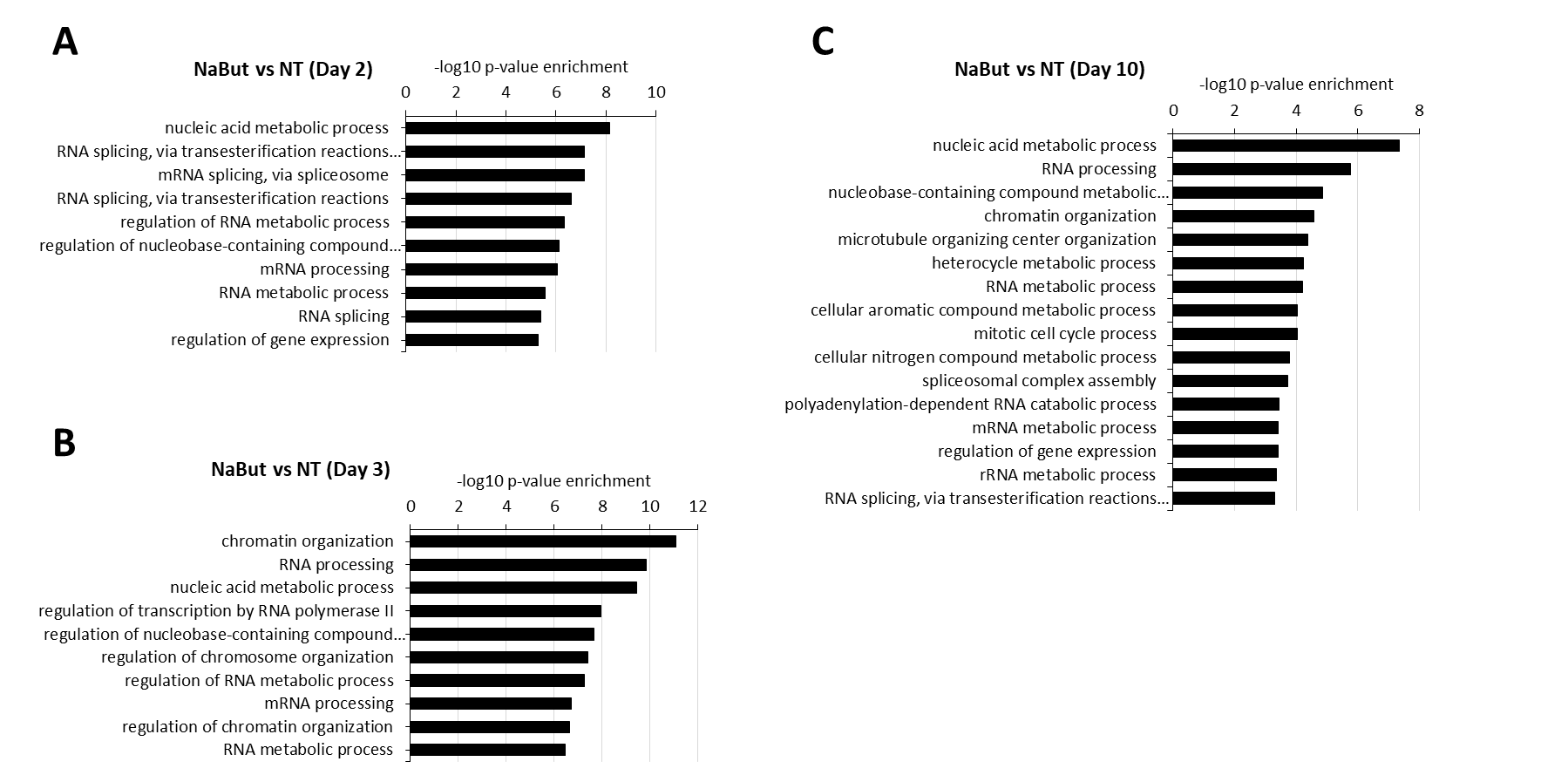
**

**Fig. S4. Nuclear functions are impaired by NaBut treatment.** Gene ontology (GO) of down regulated proteins of spheroids treated for **(A)** 2 and **(B)** 3 days as well as **(C)** after recovery (day 10). Functional annotation was obtained using GOrilla [26]. There was no significant enrichment on Day 1.

**Supplementary Table 1. Full metabolite profiling of the cell culture supernatant.**

**Supplementary Table 2. Gene expression clustering.**

**Supplementary Table 3. Proteome of HepG2/C3A spheroids and flat cells.**
